# Identification of the Relationship Between Health Professionals’ Managerial Attitudes and Distractions in the Operating Room: A Mixed Design Study

**DOI:** 10.1155/jonm/5222283

**Published:** 2026-04-12

**Authors:** Yasemin Güner, Melek Üçüncüoğlu

**Affiliations:** ^1^ Department of Nursing, Karadeniz Technical University, Trabzon, Türkiye, ktu.edu.tr

**Keywords:** distraction, managerial attitudes, nurses, operating rooms, patient safety, technician

## Abstract

**Background:**

Operating rooms are high‐risk environments where managerial attitudes and workplace distractions significantly impact patient safety and teamwork. However, the relationship between these factors remains insufficiently explored.

**Purpose:**

This study aims to identify the relationship between healthcare professionals’ managerial attitudes and distractions in the operating room.

**Methods:**

A mixed‐methods research design was employed. Quantitative data were collected using a descriptive and cross‐sectional approach, while qualitative data were obtained through a phenomenological design. 82 healthcare professionals in the operating room of a hospital were involved in the study. The Operating Room Management Attitude Scale and the Distractions in Surgery Index were used for quantitative assessment and analyzed via SPSS 22.0. Semistructured in‐depth interviews provided qualitative data, which were evaluated using content analysis.

**Results:**

Managerial attitudes were identified as key determinants of team dynamics and workplace distractions. The total mean score of the Operating Room Management Attitude Scale was 148.95 ± 37.5, with the most affected subscales being stress and fatigue (30.85 ± 7.46) and work values (28.48 ± 8.12). The most common sources of distraction included temperature (62.68%), unavailable or not working equipment (57.8%), and tiredness (68.9%). Furthermore, inadequate organizational structures, communication deficiencies, and environmental factors contributed to increased distraction levels. Leadership style directly affected employees’ job satisfaction and stress levels.

**Conclusions:**

Implications for practice: Implementing effective management strategies, ensuring a balanced distribution of workload, and minimizing distractions in the operating room can enhance patient safety by improving the efficiency and well‐being of healthcare professionals. Future research should focus on evaluating the effectiveness of targeted interventions to mitigate these challenges.

## 1. Introduction

Operating rooms are high‐intensity environments where advanced medical technologies and innovative surgical techniques are utilized, requiring effective teamwork and rapid decision‐making to ensure optimal patient outcomes [1, 2]. Research indicates that surgical team collaboration significantly influences surgical outcomes, while managerial attitudes play a critical role in shaping team efficiency and workplace dynamics [3, 4]. However, the same dynamics also make the operating rooms highly susceptible to various forms of distractions that impair concentration and disrupt the surgical flow.

Distractions are stimuli unrelated to the primary task that divert attention and lead to cognitive resource fragmentation, ultimately reducing focus and efficiency [5]. Various environmental factors, including noise, frequent staff movement, irrelevant conversations, and audiovisual stimuli, have also been identified as significant sources of distraction in surgical settings [6, 7]. One study found that an average of 56 distracting events occurred per hour during surgical procedures [8]. In another study, human‐generated noise, such as staff conversations, was perceived as more disturbing than machine‐generated noise, despite levels [9]. These findings highlight that distractions are not only frequent but also significantly detrimental to communication, workflow, and team performance.

Notably, during the surgical count, elements such as background music, personal conversations, and phone use were observed to interfere with concentration. Furthermore, intraoperative distractions, such as surgeons making additional requests and team members shifting their focus to secondary tasks, have been recognized as potential disruptors [10].

The widespread use of smartphones has introduced an additional source of distraction. Although smartphones can facilitate rapid access to clinical information, nonclinical use may reduce focus. Alshaya et al. (2025) reported that 52.8% of anesthesiologists experienced distraction related to smartphone use, despite acknowledging certain clinical benefits [11]. This dual effect reveals the growing importance of attention management in technologically advanced operating room environments.

Literature highlights the importance of teamwork, collaboration, and communication skills in the operating room [12]. In addition, managerial competencies have been reported to contribute to improved surgical outcomes. While several studies have addressed administrative challenges and distractions in surgical settings [3, 7, 10], qualitative research exploring the relationship between these factors and the experiences of healthcare professionals remains limited.

Therefore, this study aims to examine the relationship between healthcare professionals’ managerial attitudes and distractions in the operating room using a mixed‐methods approach. By integrating quantitative assessment with qualitative insights, this research seeks to provide a comprehensive understanding of how managerial factors shape distraction experiences and how these, in turn, influence teamwork and patient safety.

Purpose: In this context, the present study examined the relationship between the managerial attitudes of healthcare professionals and distractions in the operating room and their perspectives on these issues.

### 1.1. Implications of the Research

The findings of this study are expected to contribute to the understanding of how managerial attitudes influence distraction experiences and team dynamics in the operating room. By revealing both the measurable and perceived effects of management practices, this research provides evidence to guide the development of leadership strategies, structured communication practices, and organizational improvements aimed at reducing distractions. Ultimately, these insights may support the creation of safer operating room environments, enhance team performance, and improve patient safety outcomes.

## 2. Methods

### 2.1. Study Design

This study employed a mixed‐methods research design, incorporating both quantitative and qualitative data. This study was reported in accordance with the Strengthening the Reporting of Observational Studies in Epidemiology (STROBE) guidelines for cross‐sectional studies.

### 2.2. Setting and Sampling Procedure

The study population consists of healthcare professionals in two operating rooms within a hospital setting. 68 nurses and 84 surgical technicians comprised the study population. The quantitative data collection phase aimed to include the entire population, ultimately obtaining responses from 82 participants, including 36 nurses and 46 surgical technicians, who consented to participate and were accessible during the study period.

The qualitative phase of the study employed a snowball sampling method. 10 healthcare professionals, including five nurses and five surgical technicians, participated in in‐depth interviews. Data collection continued until thematic saturation was reached. In the qualitative phase, participants were selected based on having at least 10 years of operating room experience, being familiar with operating room management processes, and voluntarily agreeing to participate. The snowball technique began with initial participants recommended by the charge nurse, who then referred additional eligible participants.

The sample size was not determined through a formal power analysis, as the study aimed to include the entire accessible population of operating room staff within the study setting. Therefore, a census sampling approach was adopted, and all eligible healthcare professionals were invited to participate. A total of 82 participants who were available and consented to participate were included in the quantitative phase, which is considered adequate for descriptive and correlational analyses in similar settings.

Inclusion criteria for the quantitative phase were as follows: (1) being a nurse or surgical technician working in the operating room, (2) having at least 6 months of work experience in the operating room, and (3) willingness to participate in the study. Exclusion criteria included the following: (1) being on leave during the data collection period, (2) incomplete questionnaire responses, and (3) refusal to participate.

For the qualitative phase, inclusion criteria included having at least 10 years of operating room experience, being familiar with operating room management processes, and volunteering to participate. Participants who were unwilling to be audio‐recorded or unable to complete the interview process were excluded.

### 2.3. Data Collection

The data collection process was conducted in two stages: In the first stage, verbally informed consent was obtained from healthcare professionals willing to participate. Participants then completed the Health Professionals Descriptive Information Form, the Operating Room Management Attitude Scale, and the Distractions in Surgery Index (DiSI), which were distributed and collected by the charge nurse in the respective operating room. Quantitative data were collected between October and December 2024.

In the second stage, the charge nurse identified nurses and surgical technicians with more than 10 years of operating room experience. Individual in‐depth interviews were conducted with these participants using a structured Qualitative Interview Form. A total of 360 min of interviews were recorded. Participants were informed that audio recordings would be taken, transcribed, and analyzed, and their consent was obtained before proceeding with the interviews. Qualitative data were collected in January 2025.

The qualitative interview guide included open‐ended questions focusing on:

1. Experiences with managerial attitudes in the operating room.
2. Perceptions of distractions and their impact on workflow.
3. Communication and teamwork challenges.
4. Environmental and organizational factors contributing to distractions.
5. Suggestions for improving operating room management.

Interviews were conducted face‐to‐face in a quiet room inside the hospital, each lasting approximately 30–45 min. Audio recordings were transcribed verbatim within 24 h to ensure accuracy.

In order to conduct the study, ethics committee permission numbered 24,237,859–553 was obtained from the Karadeniz Technical University Faculty of Medicine Scientific Research Ethics Committee.

### 2.4. Instruments

Health Professionals Descriptive Information Form: Developed by the researchers, the form consists of seven items designed to assess the sociodemographic characteristics of healthcare professionals in the study.

The Operating Room Management Attitude Scale: The Operating Room Management Attitude Scale, originally developed by Helmreich and Schaefer (1995), assesses the attitudes of operating room teams toward management [13]. The scale was adapted into Turkish by Yalçınkaya (2010), with a validity and reliability study confirming a Cronbach’s alpha value of 0.82. It consists of 60 items across eight subscales: leadership, communication, information exchange, trust, stress and fatigue, teamwork, work values, error procedures, and organizational environment. Higher scores indicate more positive attitudes toward operating room management, while lower scores suggest negative perceptions [14]. In our study, the Cronbach’s alpha coefficient for the Operating Room Management Attitude Scale (ORMAQ) was 0.793.

The DiSI: The index, developed by Sevdalis et al., assesses operating room healthcare workers’ perceptions of distractions [15]. The scale was adapted into Turkish by Soyer Er and Yavuz van Giersbergen in 2024. It consists of six subscales: Individual skills, performance, and personality; operating room environment; communication; coordination/situational awareness; patient‐related disruptions; and team and organizational disruptions. Participants rate each item on a scale from 0 (*none*) to 9 (*extreme*) based on its frequency of occurrence and its perceived contribution to the potential for error. The Cronbach *α* coefficient of the scale was 0.953 for frequency, 0.967 for contribution to error, and 0.971 for obstruction of goals. [16]. In our study, the Cronbach’s alpha coefficient for the DiSI was 0.94.

### 2.5. Data Analysis

Quantitative data were analyzed using SPSS 22 statistical software. Normality was assessed using the Kolmogorov−Smirnov and Shapiro−Wilk tests. For normally distributed data, parametric tests (*t*‐test and ANOVA) were applied, while nonparametric tests (Mann−Whitney U and Kruskal−Wallis) were used for non‐normally distributed data.

Qualitative data were analyzed using the content analysis method. Initially, researchers identified codes, which were then categorized and organized into themes. To enhance rigor, two researchers independently coded the transcripts, compared codes, and resolved discrepancies through discussion. Credibility was supported through detailed field notes and repeated transcript readings.

## 3. Results

### 3.1. Quantitative Results

An analysis of the sociodemographic characteristics of the study participants revealed that the majority were female (73.2%) and married (69.5%). In terms of educational attainment, the largest group held an undergraduate degree (50%), while the distribution of professional roles was 43.9% nurses and 56.1% surgical technicians. The mean age of participants was 36.04 ± 7.2 years, and the mean duration of professional experience was 13.54 ± 7.5 years.

The total score on the Operating Room Management Attitude Scale was 148.95 ± 37.5. Among the subscales, the highest mean scores were observed in work values (28.48 ± 8.12) and stress and fatigue (30.85 ± 7.46) (Table 1).

**TABLE 1:** Mean scores of the Operating Room Management Attitude Scale and its subscales.

Scale and subscales		**x̅** ± S.D.
The Operating Room Management Attitude Scale		148.95 ± 37.5

Subdimensions	Leadership	9.94 ± 3.38
	Trust	17.51 ± 5.05
	Information exchange	11.95 ± 3.57
	Stress and fatigue	30.85 ± 7.46
	Teamwork	21.98 ± 5.55
	Work values	28.48 ± 8.12
	Error procedures	12.54 ± 4.65
	Error procedures	15.71 ± 5.46

Table 2 shows the frequency of each distraction element included in the Surgical Distractions Index, their contribution to errors, and their scores in terms of hindering the achievement of objectives. According to the DiSI, tiredness (68.9%) and lapses in attention (52.44%) were significant contributors to errors. Among environmental factors, temperature (62.68%) and unavailable or not working equipment (57.80%) were reported as major distractions. In addition, deficiencies in team communication and coordination were evident, with team members being late (38.78%) and absent during surgery (41.83%), posing potential risks to patient safety. Overall, distractions in the operating room increased error rates and were directly associated with managerial attitudes (Table 2).

**TABLE 2:** Mean scores of the Distractions in Surgery Index and its subscales.

The Distractions in Surgery Index	Frequency (%)	Contribution to error **x̅** ± S.D.	Obstruction of goals **x̅** ± S.D.
*Individuals’ skill, performance, and personality*			
Tiredness	68.9	7.24 ± 2.55	6.67 ± 3.07
Lapses in attention	52.44	6.96 ± 2.82	6.22 ± 3.17
Short‐temperedness	57.32	6.63 ± 2.80	5.89 ± 3.17
Overconfidence	58.78	6.09 ± 3.10	5.52 ± 3.33
Lack of feedback on performance	53.29	5.35 ± 3.10	5.12 ± 3.22

*Operating room environment*			
Bleeps	42.80	4.72 ± 3.19	4.66 ± 3.14
External noise	52.93	5.39 ± 3.13	5.07 ± 3.13
Loud music	41.22	4.90 ± 3.34	4.51 ± 3.14
People walking in and out of the operating room	45.37	5.22 ± 3.09	4.70 ± 3.16
Temperature	62.68	5.88 ± 2.95	6.02 ± 3.02
Unavailable or not working equipment	57.80	6.06 ± 3.01	6.00 ± 3.14

*Communication*			
Irrelevant chatting	46.46	4.48 ± 3.23	4.27 ± 3.17
Language issues	37.68	4.24 ± 3.34	4.00 ± 3.22

*Coordination and situational awareness*			
Late changes to the operating list	49.76	4.76 ± 3.33	4.83 ± 3.13
Management of the next case(s)	44.27	4.87 ± 3.23	4.79 ± 3.18
Team members being late	38.78	4.49 ± 3.31	4.72 ± 3.32
Team members being absent during the procedure	41.83	5.01 ± 3.46	5.13 ± 3.33
Lack of awareness of team process(es)	41.83	5.23 ± 3.28	5.07 ± 3.16
Multitasking	54.15	5.99 ± 3.16	5.74 ± 3.18

*Patient-related disruptions*			
Lack of necessary patient information	43.29	5.48 ± 3.47	5.15 ± 3.45
Inaccurate patient information	34.88	5.61 ± 3.53	5.28 ± 3.51
Unavailable preoperative notes	35.49	5.38 ± 3.58	5.39 ± 3.39
Unavailable test results	37.20	5.35 ± 3.56	5.33 ± 3.47

*Team and organizational disruptions*			
Not feeling part of the team	46.10	5.12 ± 3.3.43	5.34 ± 3.24
Low morale	57.32	5.71 ± 3.33	5.59 ± 3.31
Teaching	43.17	4.89 ± 3.42	4.87 ± 3.24
Time pressure	61.71	6.28 ± 3.16	6.00 ± 3.30
Hospital rationing policies	63.90	5.79 ± 3.28	5.76 ± 3.39
Unrealistic operating lists	40.12	4.91 ± 3.47	4.70 ± 3.46

The correlation analysis performed between the total score of the Operating Room Management Attitude Scale and the subdimensions of the DiSI frequency, contribution to error, and obstruction of goals revealed no significant relationship (*p* > 0.05).

The correlation coefficients were calculated as *ρ* = −0.048 (*p* = 0.670) for frequency, *ρ* = −0.050 (*p* = 0.655) for contribution to error, and *ρ* = 0.054 (*p* = 0.631) for obstruction of goals.

These results indicate that operating room management attitudes are not associated with the levels of perceived distractions among participants.

In our study, no significant differences were found between sociodemographic variables such as gender and marital status and the scale scores.

### 3.2. Qualitative Results

A total of 10 healthcare workers participated in the qualitative phase, consisting of 5 nurses and 5 surgical technicians. The participants had at least 10 years of operating room experience, with an average professional experience of 14.2 years. Their ages ranged from 32 to 48. This experienced group provided in‐depth insights into the organizational, managerial, and environmental factors that shape distractions and teamwork dynamics in the operating room.

### 3.3. Theme 1. The Effect of Distractions on Work Performance in the Operating Room

This theme reflects how sensory and cognitive load affect task performance. Healthcare professionals have identified noise, alarms, and rapid task switching as factors that distract attention and reduce situational awareness. These distracting elements not only disrupt concentration but also interrupt workflow continuity, showing a direct connection between environmental stimuli and reduced performance efficiency.

Sensory overload and perceptual disruption: Auditory attentional distraction and noise sources, extrinsic stimuli in processing, and attentional shift.

“Sometimes there is music. Loud music. Shouting. Mobbing. Mobbing is distracting. You cannot concentrate on your work (N5).”

“Alarms keep going off, phones are constantly ringing—you try to focus on something, but you just can’t. It’s distracting (T2).”

Cognitive load and decision‐making competence: The effects of time pressure, processing time constraints, and workload overload on performance.

“You’re expected to be fast. There’s barely any time between one patient leaving and the next arriving. You don’t really get a chance to prepare. Honestly, I’m not sure how safe this is for the patients (N1).”

“They expect you to do a lot of tasks that aren’t even part of your job. For example, you end up doing things that a surgical technician should be handling. When a patient leaves the room, I have to clean everything myself. That means I don’t properly get ready for the next patient (T4).”

### 3.4. Theme 2. The Impact of Managerial Factors on Operating Room Staff

Health professionals emphasized that leadership style, role clarity, and organizational structure significantly shape team functioning. It has been shown that ineffective management practices increase distraction by creating uncertainty, asymmetric workload distribution, and interpersonal tension. These managerial gaps have often been cited as underlying causes of both workflow interruptions and cognitive overload.

Leadership style and employee performance: Transformational vs. authoritarian leadership, organizational power balance, and authority distribution.

“One supervisor organizes everything perfectly, while another does the opposite—zero organization. In that environment, having fewer people working is better because when it gets too crowded, everyone starts giving orders, and things become chaotic (N2).”

“We never interfere with the nurses’ responsibilities. But here, a nurse has been assigned as the head of anesthesia, which shouldn’t be the case. The person in charge of anesthesia should be an anesthesia technician, not a nurse (T3).”

Workload distribution and organizational efficiency: Role ambiguity and job description misalignment.

“Job descriptions exist for a reason. Of course, in an emergency, everyone steps where needed, but for stable patients, each person should have a clearly defined role. If I’m not supposed to prepare medication, then I shouldn’t. And when a patient arrives, it should be clear who is responsible for cleaning the room (N4).”

### 3.5. Theme 3. The Impact of Teamwork and Communication on Patient Safety

This theme highlights that communication failures serve as both a distraction source and a patient safety risk. The findings demonstrate that hierarchical barriers, incomplete information transfer, and inconsistent adherence to safety protocols weaken interdisciplinary collaboration and impede timely decision‐making.

Team dynamics and interdisciplinary communication: Multidisciplinary teamwork, role conflicts, and implementation of patient safety protocols.

“Let’s say a patient’s vitals suddenly change during surgery. We tell the surgeon, ‘Doctor, hold on a second, the patient’s blood pressure has spiked,’ but some surgeons just ignore it. Communication problems like this put patient safety at risk (N3).”

“The surgical team can’t proceed without completing the surgical safety form. But sometimes this process feels rushed. Instead of directly confirming the patient’s name and details, they just check the information in the system and approve it (N1).”

### 3.6. Theme 4. Stress, Job Satisfaction, and Professional Resilience

Healthcare professionals described emotional exhaustion and decreased motivation as a result of constant distraction and managerial challenges. The results indicate that chronic cognitive strain and insufficient organizational support reduce job satisfaction, thereby weakening professional resilience and potentially affecting the quality of patient care.

Job satisfaction and work fulfillment: Intrinsic motivation, job satisfaction, professional burnout, and psychological strain.

“I feel happy when I know I’ve helped a patient. But if I don’t have people on the team I can trust, my motivation drops (T5).”

It’s not physical exhaustion, but by the time I get home in the evening, my brain feels completely drained. Throughout the day, I constantly have to stand up for myself, and that is truly exhausting (T1).

## 4. Discussion

This study aims to reveal the effect of managerial attitudes on levels of distraction in the operating room. Although the quantitative findings of the study did not show a statistically significant relationship between managerial attitude scores and distraction indexes, the qualitative findings indicate that leadership style, organizational clarity, and workload distribution influence how healthcare workers experience and interpret distraction. These results suggest that managerial attitudes can have indirect but meaningful effects on the operating room environment. In this context, implementing leadership development programs that promote supportive rather than authoritarian management styles may help reduce staff tension and indirectly lower the likelihood of distraction [17].

Higher scores observed in the subscales of stress and fatigue and work values indicate that perceptions of management support and working conditions affect healthcare workers. This is consistent with previous studies emphasizing that strong leadership, clear role definitions, and supportive management practices are associated with higher team motivation and enhanced job satisfaction [6, 18]. In addition, common environmental distractions identified in the study, such as temperature fluctuations and equipment failures, align with previous research showing that such factors disrupt workflow and jeopardize patient safety [8, 9]. Structured communication training (e.g., SBAR and closed‐loop communication) and equipment preparation protocols can help create a predictable and controlled work environment to reduce stress‐related and environmental distractions.

Our study revealed that organizational gaps, unclear job descriptions, and inconsistent leadership styles contribute to increased distraction by raising workload, multitasking, and communication breakdowns. Healthcare professionals frequently stated that authoritarian leadership, poorly structured workflows, and inadequate coordination lead to cognitive strain and hinder teamwork. Previous studies have shown that unstructured management practices negatively affect team performance and increase the likelihood of errors within the team [19, 20].

In the study, healthcare professionals expressed that they experienced communication problems. They specifically identified hierarchical barriers, incomplete information transfer, and inconsistent adherence to safety protocols as sources of both distraction and patient safety risk. Inadequate organizational structures contribute to workload imbalances and disrupt intrateam communication, increasing error risk by diverting healthcare professionals’ attention [21]. The literature includes studies showing that communication deficiencies during surgery can negatively affect decision‐making and increase the risk of errors [22, 23]. In addition, it has been determined that human‐generated noise, particularly irrelevant conversations, disrupts concentration and poses an obstacle to effective teamwork [9]. Addressing these issues through regular preoperative briefings and postoperative debriefings may help clarify responsibilities, improve workflow coordination, and reduce ambiguity‐related distractions.

Healthcare professionals stated that unrealistic task lists, as well as environmental and organizational stress factors such as inadequate staffing and high workload, also contribute to fatigue and decreased motivation. Several factors including emergency procedures, staff shortages, intrateam conflicts, communication failures, hierarchical structures, and stress resulting from power imbalances can exacerbate challenges within the operating room [24]. Surgical teams experience heightened stress due to intraoperative distractions, negatively impacting their decision‐making processes [25]. Studies have shown that heavy workload, time pressure, and poor managerial support increase physical and psychological stress among surgical team members [26, 27]. To strengthen communication and reduce communication‐related distractions, implementing structured communication protocols along with noise‐reduction strategies, such as limiting nonessential conversations, may be beneficial.

Overall, this study demonstrates that while managerial attitudes may not directly correlate with distraction scores quantitatively, they are perceived by healthcare professionals as critical determinants of attention, communication, and workflow. Therefore, operating room management should prioritize improving organizational clarity, supporting staff through effective leadership, and reducing systemic sources of distraction. These efforts may contribute to safer, more efficient, and more sustainable operating room environments.

### 4.1. Limitations

This study has several limitations. First, as the research was conducted in a single hospital, the generalizability of the findings is limited. Second, the cross‐sectional design prevents conclusions about causality between management attitudes and distraction levels. Third, all measurements were based on self‐report questionnaires, which may be influenced by recall bias or social desirability bias. Finally, although the sample size was adequate for analysis, future studies with larger and more diverse populations would provide a more comprehensive understanding of the topic.

## 5. Conclusions/Implications for Practice

This study examined the relationship between healthcare professionals’ managerial attitudes and distractions in the operating room. Although quantitative findings did not show a direct relationship between managerial attitudes and distraction, qualitative findings indicated that leadership style, organizational clarity, and workload distribution significantly affected employees’ attention and work experience. The results of the study indicate that authoritarian management styles, insufficient workforce, unclear roles, and environmental factors (noise, lack of equipment, and temperature fluctuations) both increase distraction and elevate stress levels among employees. These findings once again emphasize the critical role of operating room management in team coordination and creating a safe working environment.

In practice, the managerial interventions that can be implemented to improve operating room performance and reduce distractions are as follows:

• The use of structured communication tools such as SBAR and closed‐loop communication.
• Leadership development programs aimed at adopting a supportive and collaborative leadership approach.
• Noise control strategies such as reducing unnecessary conversations and adjusting alarm levels.
• Improving operating room conditions through equipment preparation checklists and environmental arrangements.
• Standardizing preoperative briefings and postoperative debriefings.
• Balanced distribution of workload and proper staffing planning.

The holistic implementation of these approaches can reduce distractions in the operating room, strengthen team cohesion, and enhance patient safety. Recognizing the impact of managerial attitudes and the work environment on attention levels will contribute to creating a safer, more effective, and supportive operating room environment. Future research in this area will provide significant contributions to the development of health management policies and the improvement of patient safety standards.

## Author Contributions

Study conception and design: Yasemin Güner, Melek Üçüncüoğlu, Data collection: Yasemin Güner, and Melek Üçüncüoğlu.

Data analysis and interpretation: Yasemin Güner and Melek Üçüncüoğlu.

Drafting of the article: Yasemin Güner and Melek Üçüncüoğlu.

Critical revision of the article: Yasemin Güner.

## Funding

There is no financial support for the study.

## Ethics Statement

Ethical approval was obtained from the relevant ethics committee prior to conducting the study.

## Conflicts of Interest

The authors declare no conflicts of interest.

## Data Availability

The data that support the findings of this study are available from the corresponding author upon reasonable request. Due to ethical and privacy considerations, the data are not publicly available.
